# Proteomic Analysis of Rta2p-Dependent Raft-Association of Detergent-Resistant Membranes in *Candida albicans*

**DOI:** 10.1371/journal.pone.0037768

**Published:** 2012-05-25

**Authors:** Lin Wang, Yu Jia, Ren-Jie Tang, Zheng Xu, Yong-Bing Cao, Xin-Ming Jia, Yuan-Ying Jiang

**Affiliations:** 1 School of Pharmacy, Second Military Medical University, Shanghai, China; 2 Department of Immunology, Tongji University School of Medicine, Shanghai, China; Université de Nice-CNRS, France

## Abstract

In *Candida albicans*, lipid rafts (also called detergent-resistant membranes, DRMs) are involved in many cellular processes and contain many important proteins. In our previous study, we demonstrated that Rta2p was required for calcineurin-mediated azole resistance and sphingoid long-chain base release in *C. albicans*. Here, we found that Rta2p was co-localized with raft-constituted ergosterol on the plasma membrane of *C. albicans*. Furthermore, this membrane expression pattern was totally disturbed by inhibitors of either ergosterol or sphingolipid synthesis. Biochemical fractionation of DRMs together with immunoblot uncovered that Rta2p, along with well-known DRM-associated proteins (Pma1p and Gas1p homologue), was associated with DRMs and their associations were blocked by inhibitors of either ergosterol or sphingolipid synthesis. Finally, we used the proteomic analysis together with immunoblot and identified that Rta2p was required for the association of 10 proteins with DRMs. These 5 proteins (Pma1p, Gas1p homologue, Erg11p, Pmt2p and Ali1p) have been reported to be DRM-associated and also that Erg11p is a well-known target of azoles in *C. albicans*. In conclusion, our results showed that Rta2p was predominantly localized in lipid rafts and was required for the association of certain membrane proteins with lipid rafts in *C. albicans*.

## Introduction

*Candida albicans* is a dimorphic opportunistic pathogen which causes a wide variety of infections ranging from relatively focused infection like superficial mycoses in generally healthy individuals to life-threatening systemic infection candidiasis in individuals with weakened immune systems [1], [2]. *C. albicans* is now recognized as one of the most common causes of bloodstream infection with high mortality rate [3], [4]. The limited availability of therapeutic arsenals makes the study of its physiology critical in order to discover new antifungal targets [5].

The plasma membrane distribution of *C. albicans* is asymmetric with respect to sphingolipids, phospholipids and ergosterol [6]. Most, if not all, of the sphingolipids are present in the outer leaflet while phospholipids are restricted to the inner leaflet [7]. The process of phospholipids and sphingolipids moving within the lipid bilayer is termed “flip-flop”, which is driven by proteins termed flippases/floppases [8]. ATP-binding cassette transporters (Cdr1p and Cdr2p) are involved in the translocation of phospholipids from the outer leaflet to the inner leaflet of the plasma membrane in *C. albicans* [9], [10]. That is because the asymmetrical distribution of phospholipids between two monolayers of plasma membrane was altered by over expression of *CDR1* and *CDR2* both in clinical and in laboratory induced azole-resistant strains of *C. albicans* [11], [12]. On the other hand, our previous study demonstrated that Rta2p might be the floppase that moved long chain bases, the backbone of sphingolipids, to outer leaflet of the plasma membrane in *C. albicans* [13]. The distribution of ergosterol appears to have a high rate of spontaneous flipping between two leaflets [14]. Due to the observed preferential interaction between ergosterol and sphingolipids, it is likely that ergosterol is abundantly present in the outer leaflet [14].

The plasma membrane of *C. albicans* is also not homogeneous in their lipid composition. There are some membrane regions that contain higher concentration of ergosterol and sphingolipids [14], [15]. These regions are more organized than the rest of the membrane due to a higher amount of saturated acid chains and are therefore called lipid rafts [14], [15]. The compositions in lipid rafts are tightly packed structures which make raft insoluble in non-ionic detergents like Triton X-100 at low temperature [16]. For this reason, lipid rafts are also named detergent-resistant membranes (DRMs). In addition to *C. albicans* [17], [18], lipid rafts have been also identified in *Saccharomyces cerevisiae* [19], [20], [21] and in *Schizosaccharomyces pombe* [22]. In all of these organisms, lipid rafts have been found to be involved in important biological processes like mating [23], cytokinesis [22] and hyphal formation [17]. Their protein compositions are heterogeneous including both transmembrane (TM) proteins and glycosylphosphatidylinositol (GPI)-anchored proteins [14]. The multi-drug resistance transporter Cdr1p, a phospholipid translocase, is predominantly localized in lipid rafts in *C. albicans* [24], [25]. Depletion of ergosterol or sphingolipids from raft regions impairs Cdr1p-mediated drug transport in a substrate- and cell-type-specific manner [24], [25], [26]. However, the relationships between Rta2p, a sphingolipids translocase, and lipid rafts remain elusive.

In the present study, we found that Rta2p is not only present in the plasma membrane but also localized in lipid rafts of *C. albicans*. Furthermore, Rta2p was found to be required for the association of certain membrane proteins with lipid rafts in *C. albicans*.

## Results

### Rta2p was unevenly present in the plasma membrane of *C. albicans*

**Figure 1:**
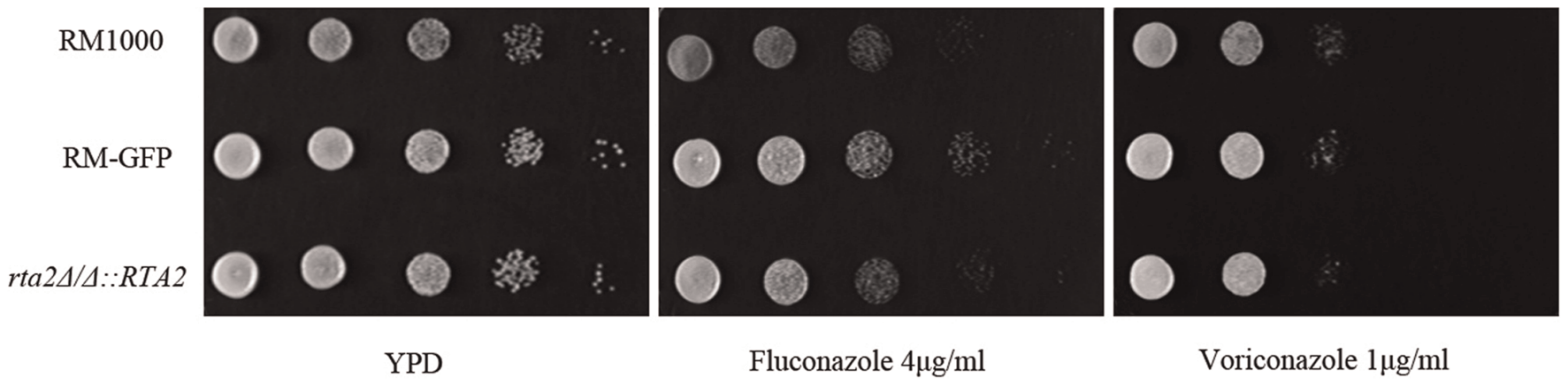
Drug susceptibility profiles of *C. albicans* strains (**Table 1**) as determined by spot assays. *C. albicans* strains including wild-type (RM1000), RM-GFP and *rta2Δ/Δ*::*RTA2* strains were spotted on YPD agar plates with or without different antifungal agents at indicated concentrations. Plates were incubated for 24 h at 35°C.

**Figure 2:**
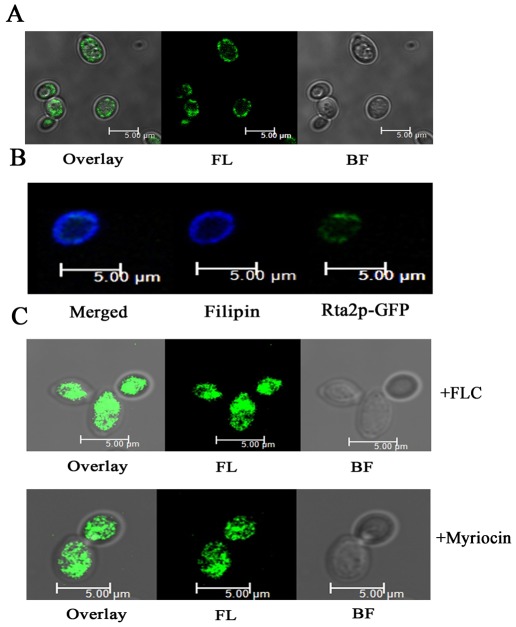
Analysis of fluorescent protein fusions in C. albicans. (**A**) Immunofluorescence mapping of Rta2p-GFP. Overlay (left), fluorescence (centre) and bright field (right) images are shown individually. (**B**) Co-localization of ergosterol-rich domains stained by filipin and Rta2p-GFP of *C. albicans*. The cells were stained with 10 µg of filipin/ml for 10 min and then analyzed by fluorescence microscopy. Merged image (left), Filipin staining (centre) and Rta2p-GFP (right). (**C**) Immunofluorescence mapping of Rta2-GFP in *C. albicans* after exposure to FLC (8 µg/ml) or myriocin (0.4 µg/ml) for 16 h. Bar, 5 µm.

Rta2p has been reported to be an integral membrane protein predicted with seven TM segments in *C. albicans* [27]. In order to explore the subcellular localization of Rta2p, we constructed the *C. albicans* strain (Table 1) wherein one *RTA2* allele was fused with the GFP-*URA3* cassette, while maintaining the native promoter sequence. The correct fusion of *RTA2* and GFP in the RM-GFP strain was verified by PCR using a primer pair specific for *RTA2* and GFP (Figure S1). It has been shown that the disruption of either one or two *RTA2* alleles in *C. albicans* displayed an increased sensitivity specifically to azoles [13]. In the present study, we found that the RM-GFP strain rendered similar sensitivities to azoles including fluconazole and voriconazole with the wild-type strain (Figure 1), which indicated that the Rta2p-GFP was functionally expressed in the transformed strain. The Rta2p-GFP fluorescence was observed to be unevenly present in the cell surface of transformed live *C. albicans* cells (Figure 2A). Furthermore, the transformed cells were co-stained with filipin, which has been used to visualize membrane sterols [28]. As evident in Figure 2B, the fluorescence of Rta2p-GFP and filipin staining were co-localized in the plasma membrane of *C. albicans*, indicating that Rta2p was an integral membrane protein.

**Table 1:** *C. albicans* strains used in this study.

Strain	Parental strain	Genotype	Reference
RM1000	RM100	*ura3Δ::imm^434^/ura3Δ::imm^434^, his1Δ::HisG/his1Δ::HisG, iro1Δ::imm^434^/iro1Δ::imm^434^*	[58]
RM-GFP	RM1000	*RM1000* * *RTA2/rta2Δ::RTA2-GFP-URA3*	This study
*rta2Δ/Δ*	JXM100	*RM1000* * *rta2Δ::hisG/rta2Δ::hisG*	[13]
*rta2Δ/Δ*::*RTA2*	*rta2Δ/Δ*	*RM1000* * *rta2Δ::hisG/rta2Δ::hisG ADE2/ade2::RTA2/URA3*	This study

* RM1000 background.

It has been well documented that ergosterol is a major constituent of lipid rafts and its biosynthesis inhibitors including fluconazole can effectively destroy the formation of lipid rafts [26]. Confocal images of fluconazole-treated *C. albicans* cells showed that Rta2p-GFP was mainly present in the intracellular compartments (Figure 2C). A similar observation was also made when myriocin was administered to inhibit the first step biosynthesis of sphingolipid [29], another major constituent of lipid rafts [30] in *C. albicans* (Figure 2C). Therefore, these data imply that Rta2p might be associated with lipid rafts in *C. albicans*.

### Rta2p was predominantly localized in lipid rafts and its association was blocked by either fluconazole or myriocin

**Figure 3:**
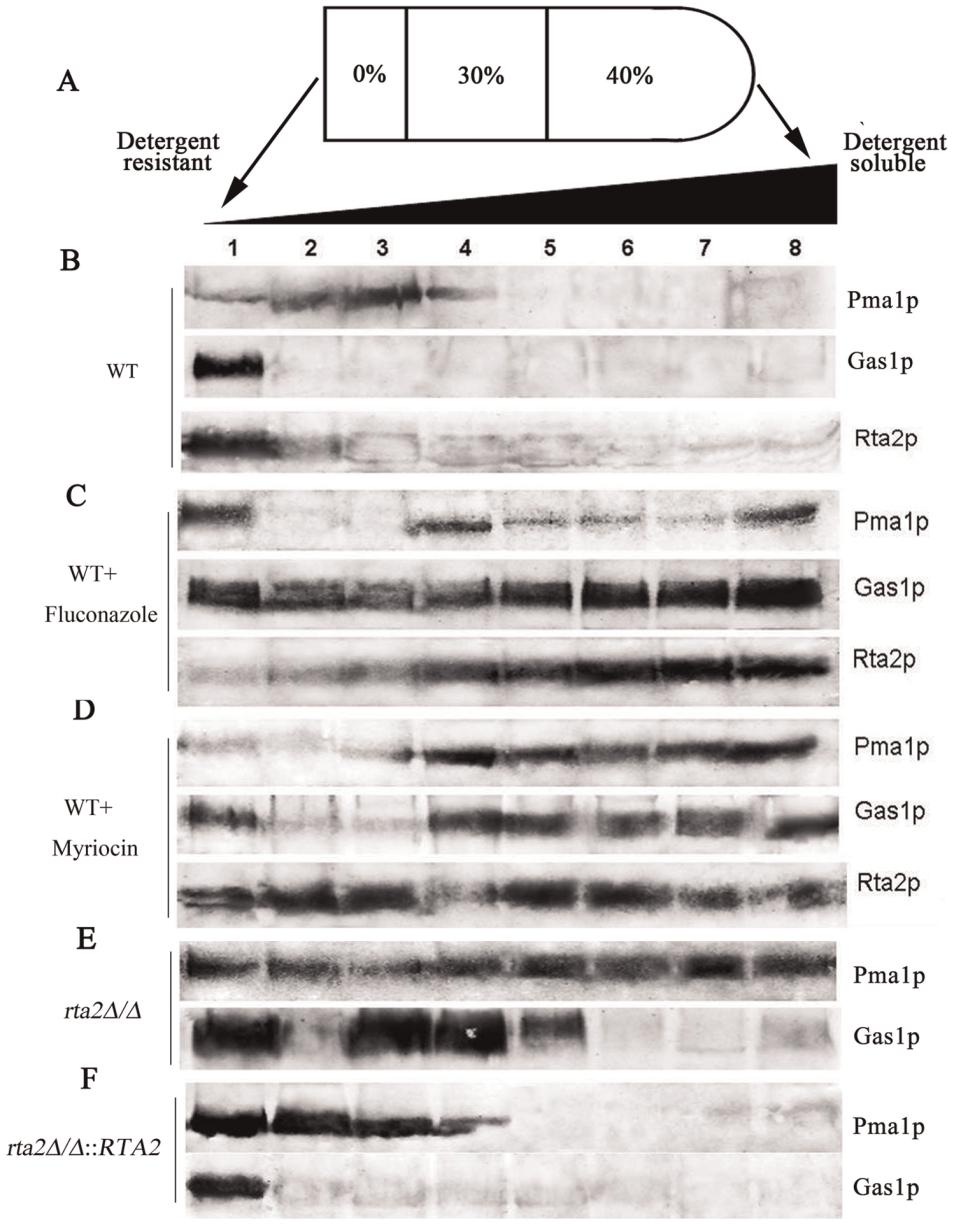
Relationships of Rta2p and DRMs in *C. albicans*. (**A**) Membranes were extracted with Triton X-100 and floated in an Optiprep gradient, as described in Materials and Methods. Fractions of equal volumes were removed from the top of the gradient and analyzed by Western blots. (**B**) Membranes proteins extracted from WT cells were analyzed on Western blots probed with antibodies against Pma1p, Gas1p homologue and Rta2p. (**C**) Membranes proteins extracted from WT cells after exposure to fluconazole (8 µg/ml) for 16 h were analyzed on Western blots probed with antibodies against Pma1p, Gas1p homologue and Rta2p. (**D**) Membranes proteins extracted from WT cells after exposure to myriocin (0.4 µg/ml) for 16 h were analyzed on Western blots probed with antibodies against Pma1p, Gas1p homologue and Rta2p. (**E**) Membranes proteins extracted from *rta2Δ/Δ* cells were analyzed on Western blots probed with antibodies against Pma1p and Gas1p homologue. (**F**) Membranes proteins extracted from *rta2Δ/Δ*::*RTA2* complemented cells were analyzed on Western blots probed with antibodies against Pma1p and Gas1p homologue. Representative data are shown from three independent experiments.

DRMs were isolated by a detergent insolubility method with the nonionic detergent Triton X-100 [31]. Optiprep gradient centrifugation was performed wherein DRM portions generally constituted the upper three fractions when eight equal fractions from top to bottom were taken (Figure 3A). The raft preparation was verified by the immunoblot with polyclonal antibodies against Pma1p and Gas1p homologue (Figure 3B), which are known to be DRM-associated in *C. albicans* [18]. Pma1p was preferentially detected in the upper three fractions and Gas1p homologue was primarily located in fraction 1 from wild-type cells (Figure 3B). Immunoblot analysis with polyclonal antibody against Rta2p clearly showed that the Rta2p was also found predominantly in fraction 1 (Figure 3B), indicating its association with DRMs.

It has been well documented that raft association of Pma1p and Gas1p homologue to the plasma membrane are dependent upon ergosterol or sphingolipid synthesis in yeast [32], [33]. We then examined the effect on the association of Rta2p with DRMs in *C. albicans* following the treatment of inhibitors (Figure 3 C and D). After exposure to fluconazole for 16 hours, the quantity of Pma1p and Gas1p homologue associated with DRMs was notably reduced and the association of Rta2p with DRMs was also blocked (Figure 3C). Following the treatment of myriocin, similar results were obtained for Pma1p, Gas1p homologue and Rta2p (Figure 3D). These results biochemically verified that Rta2p, together with well-known DRM-associated proteins (Pma1p and Gas1p homologue), was predominantly localized in DRMs and their association were blocked by either fluconazole or myriocin.

### The disruption of *RTA2* blocked the association of Pma1p and Gas1p homologue with DRMs

The localization of Rta2p in lipid rafts prompted us to investigate whether its important roles in developing fluconazole resistance were actually mediated by its regulatory effects in the formation of lipid rafts. First, we examined the effects of Rta2p on the synthesis of ergosterol in *C. albicans*. Consistent with our previous report [13], the disruption of *RTA2* had no influence on the synthesis of ergosterol (Table S1). The quantity of ergosterol from DRMs was measured by GC-MS and no significant differences were found between the wild-type, *rta2Δ/Δ* mutant and *rta2Δ/Δ*::*RTA2* complemented strains (Table 2). Spot assay showed that the *rta2Δ/Δ*::*RTA2* complemented strain rendered similar sensitivities to azoles including fluconazole and voriconazole with wild-type strain (Figure 1), which confirmed that the function of Rta2p had been restored.

**Table 2:** Percentage of ergosterol in every two fractions of the gradients from rafts to non-rafts.

strain	Percentage of ergosterol^a^			
	F_1–2_ b	F_3–4_ b	F_5–6_ b	F_7–8_ b
Wild-type	62.5	8.3	13.2	16.0
*rta2Δ/Δ*	71.7	11.0	10.2	7.1
*rta2Δ/Δ*::*RTA2*	74.7	8.2	8.4	11.6

a Ergosterol proportions varied by less than 10% in three experiments.

b Fraction 1–2 (F_1–2_), 3–4 (F_3–4_), 5–6 (F_5–6_) and 7–8 (F_7–8_) of eight equal fractions from top to bottom after Optiprep gradient centrifugation.

Next, we investigated the effects of Rta2p on the association of membrane proteins with DRMs. Unlike wild-type cells (Figure 3B), the quantity of Pma1p and Gas1p homologue associated with DRMs was greatly reduced in *rta2Δ/Δ* mutant cells (Figure 3E). We further explored the influence of restoring the function of Rta2p on DRM-associations of Pma1p and Gas1p homologue. As shown in Figure 3F, re-introduction of one *RTA2* allele into *rta2Δ/Δ* mutant cells restored the recruitment of Pma1p and Gas1p homologue to DRMs. Therefore, the disruption of *RTA2* did not interfere with the formation of lipid rafts. However, it appeared to block the association of well-known raft-associated proteins including Pma1p and Gas1p homologue with lipid rafts.

### Proteomic analysis of the DRM-associated proteins affected by the disruption of *RTA2*

**Figure 4:**
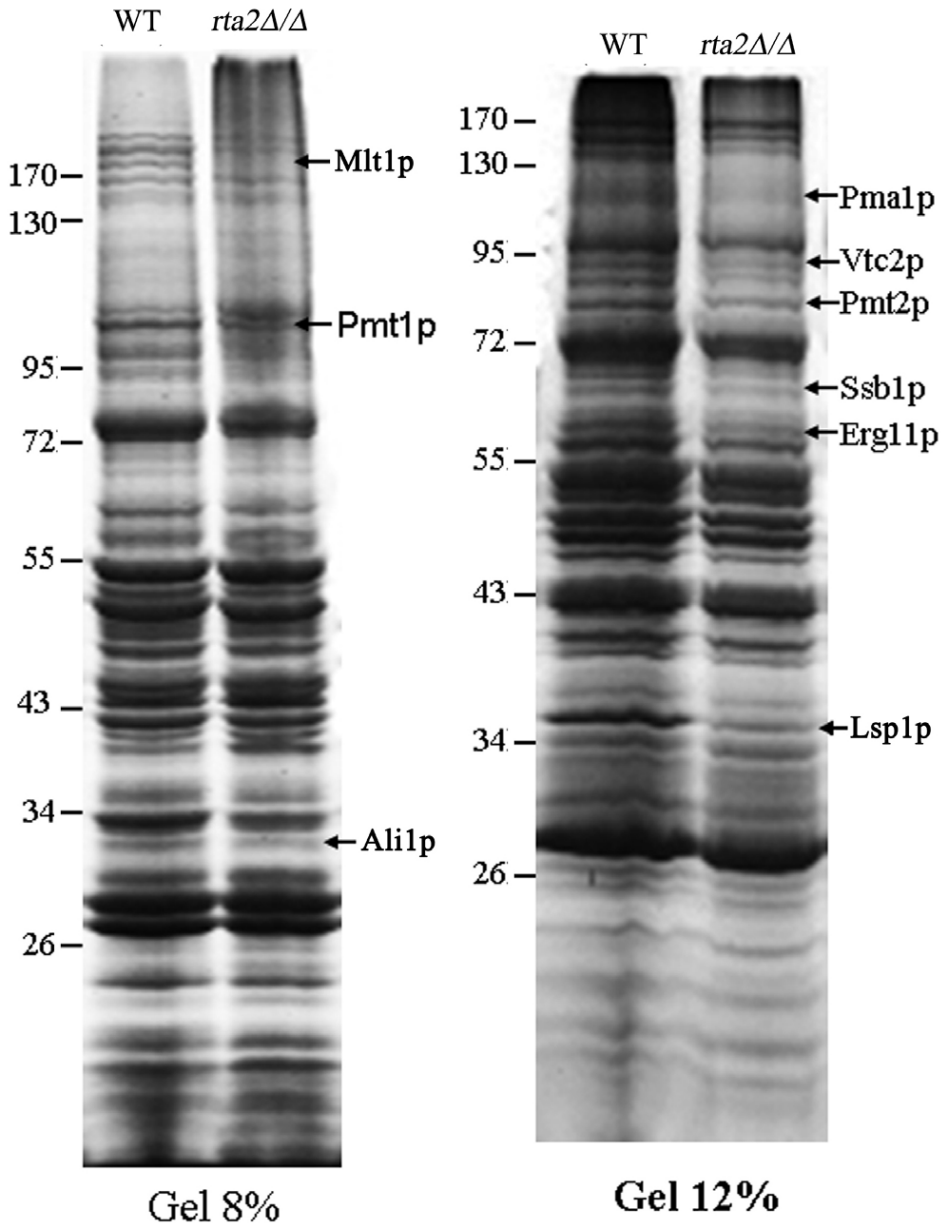
Proteomic analysis of the DRM-associated proteins affected by the disruption of *RTA2*. DRM membrane fractions, extracted from wild-type (WT) and *rta2Δ/Δ* mutant cells as described in Materials and Methods, were separated by 8% and 12% polyacrylamide gels. Representative data are shown from three independent experiments.

The influences on the association of membrane proteins with lipid rafts by Rta2p convinced us to use the proteomic analysis to identify other DRM-associated proteins, which were affected by the disruption of *RTA2*. We identified 9 differential proteins, the amount of which was significantly reduced in DRMs of *rta2Δ/Δ* mutant cells compared with wild-type cells (Figure 4). These 9 identified proteins were involved in protein modification (Pmt1p and Pmt2p), electron transport (Pma1p and Ali1p), lipid metabolism (Erg11p and Vtc2p), transporter (Mlt1p), protein binding (Ssb1p) as well as unknown functional protein (Lsp1p) (Table 3). These results implied that the disruption of *RTA2* might have blocked the association of membrane proteins with DRMs and promoted the impairment of fluconazole to the plasma membrane of *C. albicans*.

**Table 3:** DRM-associated proteins blocked by either the disruption of *RTA2* in C. *albicans*.

Protein namea)	Description	Mrb)	MPc)	%Cd)	TMe)	ANa)
Protein modification						
Pmt1p	Protein mannosyltransferase	100	30	28	11	CA4424
Pmt2p	Protein mannosyltransferase	88	20	21	8	CA5894
Electron transport						
Pma1p	Plasma membrane H(+)-ATPase	100	25	29	8	CA2300
Ali1p	Putative NADH-ubiquinone oxidoreductase	32.5	18	40		CA1557
Transporter						
Mlt1p	Vacuolar membrane transporter	180	35	18	17	CA2221
Lipid metabolism						
Erg11p	Lanosterol 14-alpha-demethylase	60.7	26	47	2	CA1387
Vtc2p	Putative polyphosphate synthetase	91.5	28	33	2	CA3918
Protein binding						
Ssb1p	HSP70 family heat shock protein	66.4	16	33		CA3534
Unknown function						
Lsp1p	sphingolipid long chain base-responsive protein	35.6	10	29		CA0622

a) Protein name and accession number according to Candida DB: http://genolist.pasteur.fr/CandidaDB.

b) Experimental *M*r (×10^−3^).

c) Number of peptide masses matching the top hit from MASCOT PMF.

d) Amino acid sequence coverage for the identified proteins.

e) TMHMM: Prediction of TM helices in proteins: http://www.cbs.dtu.dk/services/TMHMM-2.0/.

## Discussion

Rta2p has been reported to be an integral membrane protein predicted with seven TM segments in *C. albicans* [27]. Our previous study demonstrated that Rta2p, like its *S. cerevisiae* homolog Rsb1p [34], is a translocase that moves sphingolipid long chain bases from the inside to the outside of the membrane in *C. albicans* [13]. In this study, we observed that the plasma membrane distribution between Rta2p and Rsb1p was different. Rta2p, unlike its *S. cerevisiae* homolog Rsb1p, was unevenly distributed on the plasma membrane on *C. albicans*. Whereas Rsb1p had been found to be continuously and evenly distributed on membranes of cells, endoplasmic reticulum and the endosomes in *S. cerevisiae* [34]. Furthermore, the normal uneven distribution of Rta2p requires the normal synthesis of ergosterol or sphingolipid, which are known to be rich in lipid rafts [14], [15]. This disrupted distribution of membrane proteins was also observed on proteins known to be raft-associated when ergosterol or sphingolipid synthesis was inhibited [32]. The plasma membrane H^+^-ATPase Pma1p is known to be associated with DRMs [32]. Pma1p-GFP displayed a characteristic ring-like staining at the cell periphery while drug-induced inhibition of sphingolipid synthesis resulted in vacuolar localization of Pma1p-GFP in *S. cerevisiae* [30]. The raft-associated and multi-drug resistant protein Cdr1p was properly localized on the surface of wild-type *Candida* cells while the fluorescence of Cdr1p-GFP appeared to be concentrated inside the mutant cells with the sphingolipid biosynthetic gene *IPT1* disrupted [26]. Therefore, Rta2p might be like Pma1p and Cdr1p and raft-associated in *C. albicans*.

A recent proteomic study of DRMs from *C. albicans* identified 29 proteins probably localized within membrane rafts including well-known proteins associated with lipid rafts in *S. cerevisiae* [16] like Pma1p and the GPI-anchored protein Ecm33p (*S. cerevisiae* Gas1p homologue) [18]. However, Rta2p was not in the list and some known raft-associated proteins including amino acid permease Fur4p, Tat2p, and transporters Cdr1p, Can1p and Nce2p were also not identified in the proteomic analysis of DRMs from *C. albicans* [18], [35], [36]. In our study, Rta2p, along with Pma1p and Gas1p homologue, was found to be predominantly localized in lipid rafts (Figure 3B) and the raft-association of Rta2p was blocked by inhibitors of either ergosterol or sphingolipid synthesis in *C. albicans*, as determined by raft isolation (Figure 3C). Furthermore, the disruption of *RTA2* blocked the raft-association of membrane proteins (Pma1p and Gas1p homologue) and re-introduction of one *RTA2* allele into *rta2* mutant lead to the recruitment of these proteins to lipid rafts (Figure 3). Proteomic analysis revealed that, besides Pma1p, the disruption of *RTA2* inhibited the raft-association of other 8 proteins (Figure 4), ordered in Table 3 according to their function. Of these proteins, 4 (Pmt2p, Pma1p, Erg11p and Ali1p) had been found in lipid rafts [18].

The requirement of Rta2p for raft-association with Erg11p is very interesting because it is a well-known target of azoles, which results in predominance of toxic 14α-methylated sterols and inhibition of subsequent reactions of the ergosterol biosynthesis pathway in *C. albicans* [37]. Ergosterol is not only the major sterol in fungal cell membranes but also the major lipid raft-component. However, Rta2p affects neither the synthesis of ergosterol nor the distribution of ergosterol in lipid rafts (Table S1 and Table 2). Therefore, the disruption of *RTA2* does not affect the function of Erg11p, but blocks the association of Erg11p with lipid rafts.

We also identified that the raft-association of mannosyltransferases including Pmt1p and Pmt2p were required for Rta2p. Proteins from the Pmt family initiate O-mannosylation of secretory proteins. Functional analysis of the Pmt gene family in *C. albicans* indicates that Pmt isoforms have variable and specific roles for *in vitro* and *in vivo* growth, morphogenesis and antifungal resistance [38], [39]. The raft-association of a member of the HSP70 family, Ssb1p, is also identified to be required for Rta2p. Of note, a member of the HSP70 family (Ssa1p) and a heat shock cognate protein have been found in lipid rafts [18], [40], and the antibody against Ssa1p has been found in patients with systemic candidiasis [41].

In conclusion, Rta2p is predominantly localized in lipid rafts and the raft-association of Rta2p is blocked by inhibiting either ergosterol or sphingolipid synthesis. More importantly, the raft-association of certain membrane proteins is blocked by the disruption of *RTA2*. However, the mechanisms involved remain undetermined.

It has been well documented that, in *S. cerevisiae*, both TM and GPI-anchored proteins were delivered from the endoplasmic reticulum (ER) to the plasma membrane *via* lipid microdomains, and DRM-associations with either TM, such as Pma1p, Tat2p, Fur4p, and Can1p, or GPI-anchored proteins, such as Gas1p, were initiated in the ER [21], [33], [35], [42], [43], [44]. In a large scale screen, 28 genes, involved in lipid biosynthesis and vesicle transport, were identified to be required for raft-association of membrane compartment of Can1 [45], which contains the arginine transporter Can1p, two other proton symporters, Fur4p and Tat2p, and three tetraspan proteins with unknown functions, Sur7p, Fmp45p, and Ynl194c in *S. cerevisiae* [36], [46], [47]. Of note, the cytosolic protein Lsp1p of *S. cerevisiae* was colocalized with the membrane compartment of Can1 marker Sur7p [48], whereas the raft-association of *C. albicans* homolog Lsp1p was blocked by the disruption of *RTA2* in our study (Figure 4).

A recent study has demonstrated that sphingolipids are not necessary for raft-association of Pma1p but that the C26 fatty acid, which forms part of the yeast ceramide, is crucial [30]. Therefore, it is most likely that ceramide is essential for the specific transport of both TM and GPI-anchored proteins from the ER to the Golgi [49], [50], [51], [52]. The ceramide moiety consists of a sphingoid long-chain base (dihydrosphingosine and phytosphingosine) in yeast [53]. Because the ER is the site of ceramide synthesis, the generated long-chain bases must be delivered to the ER. Our previous study demonstrated that Rta2p was required for translocating long chain bases from the cytoplasmic side toward the extracytoplasmic side of the membrane [13]. Therefore, the disruption of *RTA2* may block the raft-association of certain membrane proteins by affecting the synthesis of ceramide.

## Materials and Methods

### Materials

Fluconazole was from Pfizer Inc (New York, N.Y.). Myriocin and filipin were purchased from Sigma (St. Louis, Mo.). Optiprep were also obtained from Sigma (St. Louis, Mo.). The protease inhibitors (4-[2-aminoethyl]benzenesulfonyl fluoride hydrochloride, E-64, pepstatin A, 1,10-phenanthroline) were purchased from Calbiochem (San Diego, CA). IRDye 680 goat anti-rabbit IgG was from LI-COR Biosciences (Lincoln, NE).

### *C. albicans* strains and culture media

*C. albicans* strains used in this study are listed in Table 1 and cultured in YPD (1% yeast extract, 2% peptone, 2% glucose) medium [13].

### Construction of *C. albicans RTA2*-GFP reporter strains and fluorescence assay

GFP reporter construction targeted for integration into specific *RTA2* loci was achieved using the method described previously [54]. In detail, *RTA2*-specific sequences were added to the universal primer sequences as described in Table S2 and used for PCR amplification with the plasmid pGFP-URA3 as the template to generate an integration cassette. This cassette was designed so that it could be ‘knocked into’ the correct *RTA2* locus, thereby generating *RTA2* heterozygote where one *RTA2* allele was replaced with the GFP-URA3 cassette, while maintaining the native promoter sequence. PCR products were amplified using Pyrobest polymerase (TaKaRa) and were transformed into the *ura3Δ/Δ* mutant (RM1000) by standard methods [13], yielding the strains carrying chromosomal C-terminal *RTA2*-GFP fusions.

Confocal imaging of the strain expressing Rta2p-GFP was performed under an oil immersion objective at ×100 magnification under a confocal microscope (Leica TCS SP5). For colocalization studies, *C. albicans* strain expressing *RTA2*-GFP was stained with 10 µg/ml filipin for 10 min and then analyzed by fluorescence microscopy [17].

### Construction of *RTA2* revertants

All the primer sequences are listed in Table S2. The fragment containing ORF of *RTA2* and its 1 kb up/downstream were amplified by PCR with Pyrobest polymerase (TaKaRa). The *PstI-KpnI* digested PCR fragments were ligated into plasmid pBes116 [55] to obtain plasmids pBes-RTA2. And then, the *AscI* digested fragments of pBes-RTA2 were transformed into *rta2Δ/Δ* mutant as before [13]. Re-introduction of one *RTA2* allele into *rta2Δ/Δ* mutant at the *ade2* locus was verified by PCR (Figure S2). The up-regulation of *RTA2* in the *rta2Δ/Δ*::*RTA2* complemented strain by calcium signaling was verified by quantitative RT-PCR (Figure S2).

### Susceptibility testing

Drug sensitivity was assayed on YPD agar plates containing drugs at the indicated concentrations as described previously [13]. DMSO, the drug vehicle, was ≤0.5% in all cases. Five microliters of tenfold serial dilutions of each yeast culture (OD_600_ = 1.0) was spotted on the appropriate medium plates and then incubated at 35°C for 24 h.

### DRM isolation and Western blot analysis

The isolation of detergent-resistant membranes (DRMs) was performed as described previously [56]. Briefly, a logarithmically growing *C. albicans* culture in YPD (OD_600_ = 1) was collected by centrifugation and lyophilized. Biomass dry weight was determined. Cell pellet was resuspended in 500 µl cold TNE buffer (50 mMTris-Cl, pH7.4, 150 mM NaCl, 5 mM EDTA) plus protease inhibitors (final concentrations of 0.2 mM 4-[2-aminoethyl]benzenesulfonyl fluoride hydrochloride, 3 µM E-64, 4 µM pepstatin A, 1 mM 1,10-phenanthroline (Calbiochem), and broken by vortexing with a 0.5 volume of glass beads for 6 cycles (30 s of vortexing followed by 30 s on ice). Unbroken cells and beads were removed by centrifugation for 15 min at 500× g. Total membranes were collected by centrifugation (30 min at 58,000× g) and resuspended in 50 µl of TNE buffer. Total quantity of protein was at this stage determined by the Bradford method. A defined amount of total protein (2.5 mg) was transferred to a microtube and added 450 µl of TNE buffer. Triton X-100 was added to a final concentration of 1%, and the mixture was incubated for 30 min on ice. The chilled supernatant fluid (465 µl) was adjusted to 40% Optiprep by adding 930 µl Optiprep solution (Sigma) and overlaid with 2.2 ml 30% Optiprep in TXNE (TNE plus 0.1% Triton X-100) and 370 µl TXNE. The samples were centrifuged at 200 000×g for 4 h in a P55ST2 rotor (Hitachi). Eight fractions of equal volume (460 µl) from top to bottom were collected, and proteins were precipitated by adding two volumes of 15% trichloroacetic acid (final concentration 10%) for 30 min on ice. Precipitated proteins were collected by centrifugation in a microcentrifuge for 20 min at 4°C, and the pellet was dissolved in 15 µl of 1 M Tris base and 35 µl of dissociation buffer (0.1 M Tris-Cl, pH 6.8, 4 mM EDTA, 4% SDS, 20% glycerol, 2% 2-mercaptoethanol, 0.02% bromphenol blue). Samples were heated at 37°C for 15 min.

SDS-PAGE was carried out on 8% polyacrylamide gels. The following antibodies were used for immunoblotting: rabbit anti-Gas1p (a gift from H. Riezman, University of Basel, Switzerland) at 1∶5000, rabbit anti-Pma1p (a gift from Guenther Daum Institute of Biochemistry Graz University of Technology) at 1∶5000 and rabbit anti-Rta2p at 1∶500. Immuno-reactive bands were detected using Infrared IRDye-labeled anti-rabbit IgGs (LI-COR) and an infrared imaging system (Odyssey).

### MS analysis and database searches

DRMs were separated by 8% and 12% polyacrylamide gels and stained with Coomassie blue G dye. The gel bands of visual difference between the wild-type and *rta2Δ/Δ* mutant cells were manually excised and subjected to in-gel trypsin digestion and subsequent MALDI-TOF/TOF identification as described previously [57]. Combined MS and MS/MS spectra were submitted to MASCOT (V2.1, Matrix Science, London, U.K.) by GPS Explorer software (Applied Biosystems) and searched with the following parameters: NCBI nr database, taxonomy of fungi, trypsin digest with one missing cleavage, none fixed modifications, MS tolerance of 0.1 Da, MS/MS tolerance of 0.8 Da, and possible oxidation of methionine. Known contaminant ions (keratin) were excluded. MASCOT protein scores (based on combined MS and MS/MS spectra) of greater than 72 were considered statistically significant (*p*≤0.05). The individual MS/MS spectrum with a statistically significant (confidence interval >95%) best ion score (based on MS/MS spectra) was accepted. To eliminate the redundancy of proteins that appeared in the database under different names and accession numbers, the single-protein member belonging to the species Gallus or else with the highest protein score (top rank) was singled out from the multi-protein family.

### Sterol analysis

Samples preparation, sterols isolation, sterol identification, and sterols analysis were done as described previously [13].

## Supporting Information

Figure S1 **Verification of the correct fusion of** ***RTA2*** **and GFP in** ***C. albicans*** **.** The *C. albicans* strain (RM-GFP) carrying chromosomal C-terminal *RTA2*-GFP fusions, yielded one 1.79 kb PCR product by PCR analysis with the forward primer specific to *RTA2* and the reverse primer specific to GFP, with the wild-type strain (RM1000) as control.
(JPG)

Figure S2 **Verification of the re-introduction of one** ***RTA2*** **allele into** *rta2* ***Δ/Δ*** **mutant.** (**A**) The *rta2Δ/Δ*::*RTA2* complemented strain (JXM201), with one allele of *RTA2* reintroduced into *ADE2* locus, yielded only one 3.8 kb PCR product by PCR analysis with the forward primer to *ADE2* and the reverse primer specific to *RTA2*, with the plasmid pBes-RTA2 as control. (**B**) Expression levels of *RTA2* were examined by quantitative RT-PCR in the wild-type strain (RM1000) and the *rta2Δ/Δ*::*RTA2* complemented strain (JXM201) after exposure to 1 mM CaCl_2_ for 16 h, with their corresponding drug-free strains as controls.
(JPG)

Table S1 The content of sterol compositions from *C. albicans* wild-type and *rta2Δ/Δ* mutant strains.
(DOC)

Table S2 Primers used in this study.
(DOC)
