# Supplementary figures and images for: Proteomic Analysis of Rta2p-Dependent Raft-Association of Detergent-Resistant Membranes in Candida albicans
Source: PLoS One. 2012 May 25;7(5):e37768. doi: 10.1371/journal.pone.0037768 (PMC3360622; doi:10.1371/journal.pone.0037768)

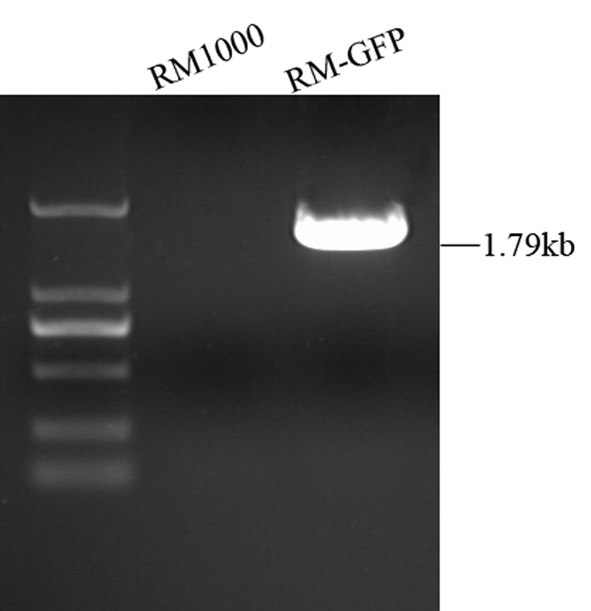

Supplement: Figure S1 — Verification of the correct fusion of RTA2 and GFP in C. albicans . The C. albicans strain (RM-GFP) carrying chromosomal C-terminal RTA2-GFP fusions, yielded one 1.79 kb PCR product by PCR analysis with the forward primer specific to RTA2 and the reverse primer specific to GFP, with the wild-type strain (RM1000) as control. (JPG) [file pone.0037768.s001.jpg]

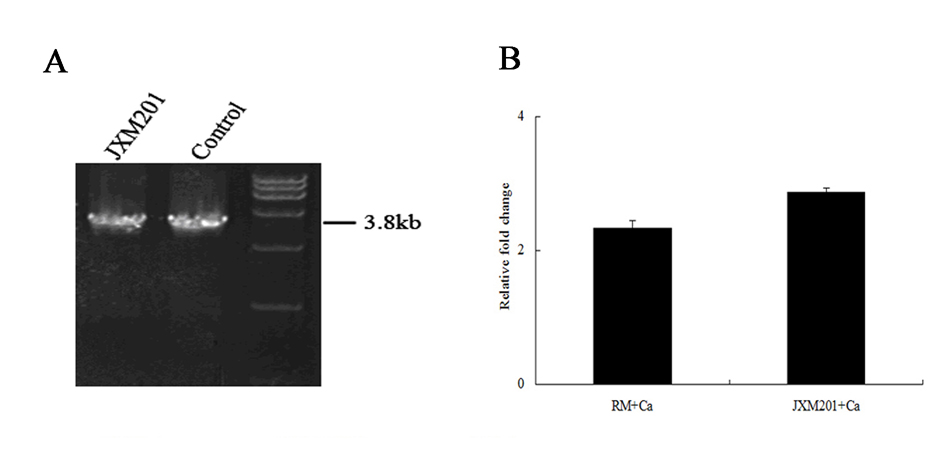

Supplement: Figure S2 — Verification of the re-introduction of one RTA2 allele into rta2 Δ/Δ mutant. (A) The rta2Δ/Δ::RTA2 complemented strain (JXM201), with one allele of RTA2 reintroduced into ADE2 locus, yielded only one 3.8 kb PCR product by PCR analysis with the forward primer to ADE2 and the reverse primer specific to RTA2, with the plasmid pBes-RTA2 as control. (B) Expression levels of RTA2 were examined by quantitative RT-PCR in the wild-type strain (RM1000) and the rta2Δ/Δ::RTA2 complemented strain (JXM201) after exposure to 1 mM CaCl2 for 16 h, with their corresponding drug-free strains as controls. (JPG) [file pone.0037768.s002.jpg]
