# Supplementary material for: Proteomic Analysis of Rta2p-Dependent Raft-Association of Detergent-Resistant Membranes in Candida albicans
Source: PLoS One. 2012 May 25;7(5):e37768. doi: 10.1371/journal.pone.0037768 (PMC3360622; doi:10.1371/journal.pone.0037768)
Supplement: Table S1 — The content of sterol compositions from C. albicans wild-type and rta2Δ/Δ mutant strains. (DOC) [file pone.0037768.s003.doc]

**Table S1** The content of sterol compositions from *C. albicans* wild-type and ***rta2****Δ/Δ* mutant strains

| strain | Sterol(μg/ml) ^a^ | | |
| --- | --- | --- | --- |
|  | Ergosterol | Lanosterol | Ergosta-4,6,8(14),22-tetraen-3-one |
| Wild-type | 333.2 | 7.2 | 21.3 |
| ***rta2****Δ/Δ* | 303.8 | 31.5 | 44.1 |

^a^ The content of *s*terol varied by less than 10% in three experiments.
