# Supplementary material for: Proteomic Analysis of Rta2p-Dependent Raft-Association of Detergent-Resistant Membranes in Candida albicans
Source: PLoS One. 2012 May 25;7(5):e37768. doi: 10.1371/journal.pone.0037768 (PMC3360622; doi:10.1371/journal.pone.0037768)
Supplement: Table S2 — Primers used in this study. (DOC) [file pone.0037768.s004.doc]

**Table S2**. Primers used in this study

| Primer | sequence | |
| --- | --- | --- |
| **Primers for construction of the *RTA2* revertant strain** | | |
| RTA2-FWD | AAAACTGCAGGGTGGTATGATTCCTAGCAAC | |
| RTA2-RV | CGGGGTACCTCATCAGTATCAGCGGTGAG | |
| **Primers for verifying the integration of intact *RTA2* in the *ADE2* site** | | |
| ADE2chk-FWD | TCTCAGTCAACCATTTTGAGTC | |
| RTA2chk-RV | GTAGGTGTGGAAGTTGGAGTCC | |
| **Primers for** **construction of strain with chromosome-integrated fluorescence tags.** | | |
| RTA2-GFP-FWD | GTATTTGGTAAAAACAATATTGTCAAGTTGGCAACCATCAAGAAGAATCTTGATGAAAATGAAAAAAATCGGTGGTGGTTCTAAAGGTGAAGAATTATT | |
| RTA2-GFP-RV | CTATTTAAACAATGTATGTCATTTGCAACCACGCTAGAACTGCGTTCTTGTGATTCAACATCATCATTATATTCTAGAAGGACCACCTTTGATTG | |
| **Primers for verifying the integration of GFP in the *RTA2* site** | |  |
| RTA2chk-FWD | ATTCACGGGAGACAATATTCAG |  |
| GFPchk-RV | GCACTCACGTAAACACTTAATC |  |

The underlined sequence corresponds to the restriction enzyme site,and the overlap sequence are in bold.
